# Dynamic Expression and Functional Implications of the Cell Polarity Gene, Dchs1, During Cardiac Development

**DOI:** 10.3390/cells14110774

**Published:** 2025-05-24

**Authors:** Kathryn Byerly, Cayla Wolfe, Hannah Parris, Charlotte Griggs, Emily Wilson, Matthew Huff, Molly Griggs, Jordan Morningstar, Lilong Guo, Fulei Tang, Jan Guz, Taylor Petrucci, Ranan Phookan, Brian Loizzi, Cortney Gensemer, Russell A. Norris

**Affiliations:** 1 Department of Regenerative Medicine and Cell Biology, Medical University of South Carolina, Charleston, SC 29407, USA; byerlyk@musc.edu (K.B.); wolfecay@musc.edu (C.W.); parrihan@musc.edu (H.P.); griggsch@musc.edu (C.G.); wilemil1@musc.edu (E.W.); huffmat@musc.edu (M.H.); griggsma@musc.edu (M.G.); morningj@musc.edu (J.M.); guol@musc.edu (L.G.); petrucct@musc.edu (T.P.); phookan@musc.edu (R.P.); loizzi@musc.edu (B.L.); gensemer@musc.edu (C.G.); 2 Department of Comparative Medicine, Medical University of South Carolina, Charleston, SC 29407, USA; fulei@musc.edu (F.T.); guzj@musc.edu (J.G.); 3 Department of Neurosurgery, Medical University of South Carolina, Charleston, SC 29407, USA

**Keywords:** Dachsous, cadherin, cardiac development, cell polarity

## Abstract

Intercellular interactions among cardiac cell populations are essential for cardiac morphogenesis, yet the molecular mechanisms orchestrating these events remain incompletely understood. Dachsous1 (Dchs1), an atypical cadherin linked to mitral valve prolapse, is a core planar cell polarity protein whose function in the developing heart has not been fully elucidated. To address this, we generated a Dchs1-HA knock-in mouse model to define its spatial, temporal, and cellular expression patterns. Using immunohistochemistry, western blotting, and single-cell transcriptomics across developmental stages, we demonstrate that cardiac Dchs1 expression is restricted to non-cardiomyocyte lineages. DCHS1 displays dynamic subcellular localization and tissue organization depending on the developmental timepoint, with staining being found in epicardial and endocardial surfaces at earlier embryonic stages and in the compact myocardium in later fetal and neonatal stages. During fetal and neonatal stages, DCHS1-positive non-myocyte, non-endothelial cells form polarized extensions that bridge endothelial and non-myocyte, non-endothelial cells, suggesting direct heterotypic and homotypic interactions. Western blotting revealed evidence of DCHS1 proteolytic cleavage, with intracellular C-terminal fragments. RNA co-expression with its binding partner FAT4 supports a conserved, non-myocyte-specific DCHS1-FAT4 signaling axis. These findings identify DCHS1 as a potential molecular tether that is utilized in intercellular communications during cardiac development, with implications for congenital and acquired heart disease.

## 1. Introduction

Cellular interactions are fundamental to signaling in complex organisms and are critical to life. In the context of organ development, dynamic homotypic and heterotypic cellular interactions are required for normal morphogenetic signaling. The development of the heart, for example, relies on the precise coordination of interactions among various cell types, including cardiomyocytes, endothelial cells, and fibroblasts. Disruptions in these interactions are implicated in numerous cardiac conditions, such as congenital heart defects (e.g., hypoplastic left heart syndrome [1] and tetralogy of Fallot [2]), arrythmias (e.g., atrial fibrillation [3]), hypertrophic cardiomyopathy [4], and others. The prevalence of cardiac conditions stemming from disrupted cell–cell interactions underscores the critical role of intercellular signaling in heart development and disease.

Despite significant advances in understanding cardiac development, many questions remain about how interactions between distinct cellular populations drive lineage specification and morphogenesis. For instance, while homotypic myocyte communication [3,5,6] has been extensively studied, the roles of heterotypic myocyte-to-non-myocyte and non-myocyte-to-non-myocyte interactions in shaping cardiac development remain poorly defined. Thus, substantial gaps persist in understanding how cell organization, migratory behaviors, and mechanical forces contribute to these processes. An emerging area of interest is the role of planar cell polarity (PCP) in cardiac development, which refers to the coordinated alignment of cells within the plane of a tissue. PCP has been extensively studied in various developmental and evolutionary contexts, such as Drosophila wing morphogenesis, gastrulation and convergent extension, neural tube closure [7], gut development [8], and hair follicle orientation [9]. While the canonical PCP pathway, involving components such as Wnt, Frizzled, and Dishevelled genes, has been extensively researched [7,8,9,10,11,12], the non-canonical components of the PCP pathway are less understood. Among these non-canonical elements are large, atypical cadherin gene families, including Dachsous (Dchs) and Fat. These families encompass six key genes (Dchs1, Dchs2, Fat1, Fat2, Fat3, and Fat4) that encode large single-pass transmembrane cadherins. These cadherins direct cell–cell interactions through heterotypic binding between DCHS and FAT proteins. In tissues where these genes are co-expressed, DCHS-FAT pairs are thought to act as ligand-receptor complexes that regulate PCP and cellular differentiation decisions across various developmental contexts, including the kidney [13], brain [14,15], gut [8], inner ear, and appendicular skeleton [14,16]. Given their broad expression and essential roles in developmental processes, mutations in DCHS1 and FAT4 have been linked to numerous human diseases [17,18,19,20,21], many of which are associated with disruptions in cell polarity during morphogenesis. These findings underscore the pivotal roles of DCHS1 and FAT proteins not only in regulating morphogenesis but also in contributing to the etiology of human diseases.

The majority of research on PCP protein function has focused on tissues outside the heart. However, the discovery that haploinsufficient DCHS1 variants are a causal factor in mitral valve prolapse (MVP) [17,18,19], a common cardiac condition, combined with findings that nearly all Dchs1 knockout mice die at birth with cardiac abnormalities, provides a rationale for exploring its role in cardiac development. This study aims to define the spatial and temporal expression patterns of Dchs1 and its binding partners during cardiac morphogenesis, with the goal of providing new insights that could be linked to congenital heart defects and postnatal cardiac diseases.

## 2. Materials and Methods

### 2.1. DCHS1-HA Mouse Studies

A knock-in mouse strain was constructed by the Transgenic Genome Editing Core at the Medical University of South Carolina via CRISPR-Cas9 in which three hemagglutinin (HA) tags were inserted after exon 21 of Dchs1 (Figure S1). Briefly, a single guide RNA (sgRNA; synthesized by Synthego Corp., Redwood City, CA, USA) with the sequence 5′-CAGAGCTGCGCATCTAGCTG was designed to target the C-terminus of the murine Dchs1 locus. Homology-directed repair (HDR) was achieved by co-electroporating the sgRNA/Cas9 ribonucleoprotein (RNP) complex together with a single-stranded oligodeoxynucleotide (ssODN; synthesized by IDT, Coralville, IA, USA) containing the 3xHA tag sequence into C57BL/6J zygotes (Jackson Laboratory, Bar Harbor, USA, Stock #00064) using a NEPA21 electroporator (Sonidel, Boston, MA, USA). The electroporation settings were: “Poring pulse”: 175 V, 1 ms pulse width, 50 ms interval, 4 pulses; and “Transfer pulse”: 20 V, 50 ms pulse width, 50 ms interval, 5 pulses (±polarity, 40% attenuation rate). The embryos that developed into the two-cell stage were selected and transferred into the oviducts of pseudo-pregnant female mice. F0 founders were screened by tail biopsy genotyping, and potential knock-in alleles were identified via PCR amplification of the Dchs1 locus using primers 5′-CTCTCCCGTTGTCTCACCAT and 5′-TTTAAGGTGGAGAGCAGGGG. In-frame integration of 3 repeated HA tags was confirmed by Sanger sequencing of PCR products. The correctly targeted founders were bred with C57BL/6J mice, followed by PCR genotyping and Sanger sequencing to confirm the germline transmission of the inserted sequences. The resultant mouse strain C57BL/6J-Dchs1em1(HA) (termed Dchs1-HA) allowed antibody-based analysis of the resultant DCHS1-HA protein (with the HA-tag on the C-terminal end) due to a lack of consistent antibodies against DCHS1. Heterozygous mice with the HA tag (termed Dchs1HA/+) were crossed with C57BL/6J mice. Pups from the resultant litters had their hearts excised for western analyses and immunohistochemistry with a minimum n = 3 used for each timepoint analyzed via each method. Hearts were excised from embryonic day 11.5 (E11.5), E13.5, E18.5, post-natal day 0 (P0), P7, and P21 litters. These timepoints were chosen to reflect major developmental milestones in cardiac development. At E11.5, the epicardium has invested around the myocardium [22], and by E13.5, epicardial-derived cells have begun migrating through the developing ventricular free wall [23]. By E18.5, septation is completed, and all major cardiac structures have formed [24]. Birth causes a switch from fetal to adult circulation, which is captured by the selected P0 timepoint. The adolescent period is captured by P7 and P21, at which point all major cardiac cell lineages have fully differentiated [25]. All animal experiments were performed under protocols approved by the Institutional Animal Care and Use Committees at the Medical University of South Carolina. Prior to tissue biopsy, mice were euthanized by isoflurane (Piramal) induction, followed by cervical dislocation in line with the Guide for the Care and Use of Laboratory Animals (NIH publication no. 85–23, revised 1996). For embryonic, fetal, and neonatal timepoints (E11.5–P0), the pups were sacrificed via decapitation. For the adolescent timepoints (P7 and P21), the mice were anesthetized with isoflurane; the P7 mice were euthanized via decapitation, and the P21 mice were euthanized via cervical dislocation. All animal experiments were performed in accordance with IACUC procedures and approved protocol number IACUC-2020–00956 (Initial Approval date: 23 April 2020; Last Approval Date: 27 January 2025; Expiration Date: 7 March 2026). There are no human subjects involved in the study; thus, no relevant IRB approvals are needed.

### 2.2. Western Analyses

After euthanasia at all timepoints, the hearts were excised, digested in radioimmunoprecipitation assay (RIPA) buffer, minced with spring scissors, sonicated, and centrifuged (10 min at 17,000× *g* at 4 °C). Protein lysates were probed by western blotting and blinded to genotype as we have previously reported [18]. The primary antibody used for western analysis was an α-HA (Invitrogen #MA5-27915; Waltham, MA, USA) at a 1:1000 dilution, and the secondary antibody was a goat α-Rabbit IgG-HRP (Sigma #A8275; Saint Louis, MO, USA). Bands were detected by SuperSignal West Fempto Substrate (Pierce # 34096; Waltham, MA, USA).

### 2.3. Immunohistochemistry

After euthanasia and excision via the same procedures as above, hearts were fixed in 4% paraformaldehyde for 4 h at room temperature, dehydrated, and processed through graded alcohols and toluene, paraffinized, embedded in paraffin, and sectioned onto charged slides. Slides were then stained through rehydration in xylene and graded alcohols, and epitopes were exposed through 1 min pressure cook at high pressure in a 0.93% citric acid solution (Vector Laboratories, #H-3300; Newark, NJ, USA). The exposed tissue was blocked with 1% bovine serum albumin (Invitrogen, # B4287) for 1 h at room temperature, probed with primary antibody for 1 h at room temperature, washed with 1X phosphate buffered solution (PBS), probed with secondary antibody for 1 h at room temperature, washed with 1X PBS with 0.0001% Hoechst (Invitrogen, # H3569, *v*/*v*), and cover-slipped with Thermo Slow Fade (Invitrogen, # S36936). The following primary antibodies and reagents were used for immunostaining: rabbit anti-HA antibody (Cell Signaling, # 3724; Danvers, MN, USA) at a dilution of 1:250, rat anti-CD31 antibody (Dianova, # DIA-310; Hamburg, Germany) at 1:100, and mouse anti-MF20 antibody (Developmental Studies Hybridoma Bank, Concentrate 0.1 mL; Iowa City, IA, USA) at 1:500. 

### 2.4. RNA Sequencing

Five wildtype P0 mice were euthanized via the same procedures as above, and isolated hearts were snap-frozen in liquid nitrogen. For nuclei isolation, the hearts were minced with spring scissors in lysis buffer (10 mM Tris-HCl pH 7.0 Invitrogen # AM9850G, 10 mM NaCl Invitrogen # AM9760G, 3 mM MgCl_2_ Ambion; Waltham, MA, USA # AM9530G, 0.1% Tween-20 Sigma # P9416, 0.1% NP40 Thermo # J60766.AP, 0.01% Digitonin Invitrogen # BN2006, 1% Bovine Serum Albumin Sigma # A1595, 1 mM Dithiothreitol BioRad # 16160611, 2 U/μL RNAse Inhibitor Thermo # N8080119), dissociated via shaking at 300 rpm at 4 °C for 5 min, and homogenized using a 2 mL dounce (Sigma # P1110). Nuclei were then isolated via centrifugation at 2000× *g* for 5 min at 4 °C and purified via centrifugation in graded strainers (CellTrics 100 µm # 04-004-2328 and CellTrics 20 µm # 04-004-2325) and resuspended in wash buffer (10 mM Tris-HCl pH 7.0 Invitrogen # AM9850G, 10 mM NaCl Invitrogen # AM9760G, 3 mM MgCl_2_ Ambion # AM9530G, 0.1% Tween-20 Sigma # P9416, 1% Bovine Serum Albumin Sigma # A1595, 1 mM Dithiothreitol BioRad (Hercules, CA, USA) # 16160611, 2 U/μL RNAse Inhibitor Thermo # N8080119). The nuclei pellet was resuspended in nuclei buffer (1% Bovine Serum Albumin Sigma # A1595, 2 U/μL RNAse Inhibitor Thermo # N8080119, Dulbecco’s Phosphate Buffered solution ATCC # 30-2200), and nuclei concentration determined using trypan blue exclusion (Trypan Blue stain 0.4% Cat. T10282) and processed for cDNA library preparation by 10X Chromium Next GEM Single Cell 3′ Reagent Kits V3.1 (Dual Index) per manufacturer’s protocol. Quality control of the library prep was confirmed by Bioanalyzer and NextGen sequencing was performed by Azenta, Inc; Burlington, NJ, USA.

FASTQ files were aligned to the mouse genome (version Mm10) and counted using 10X Genomics Cell Ranger (version 8.0.1)’s “count” command. Ambient RNA contamination was predicted and removed using Cell Bender (version 1.0.3) with default options [26]. The Cell Bender results were read as input in Seurat (version 5.1.0) [27]. The percentage of mitochondrial genes was calculated by comparing the number of genes beginning with “Mt”, or any variations thereof, against the total number of genes in each cell. Cells containing 5% or more mitochondrial genes or less than 250 unique molecular identified (UMI) counts were filtered out. Following filtering, the data in the Seurat object were split using the SplitObject command, transformed using the SCTransform command, and integrated. Integration features were identified from 18,880 gene features prior to data integration. Following integration, a principal component analysis (PCA) was run on the data. Batch effects were corrected using Harmony (version 1.2.1) [28] and clustered with a resolution of 0.6. These clustered data were converted to a single-cell experiment object using SingleCellExperiment (version 1.26.0) [29] to identify singlets and doublets through ScDblFinder (version 1.18.0) [30]. Only cells labeled “singlets” were kept in the Seurat object, and the same clustering workflow described above was rerun on the filtered dataset. Each cluster was then assigned a cell type based on gene expression. This step was performed manually by comparing expression of key marker genes through a dot plot. We focused on genes associated with a single-cell profile of genes associated with heart cells [31]. Cardiomyocyte types, including nodal cells, were differentiated using an additional set of genes associated with specific cardiomyocyte types.

For additional timepoints to investigate RNA Dchs1 expression analysis, single-cell RNA sequencing datasets for wild-type E12.5 and E16.5 murine hearts were downloaded from existing BioProject PRJNA890252 [32] and probed for Dchs1. The same pipeline that was run on the P0 cells was run on these samples. Due to the lower read quality of these samples compared to the P0 samples, the reads were trimmed with a minimum read length of 30 bp and a minimum quality score of 30 using Skewer (version 0.2.2) [33]. Lastly, 3-month-old murine heart single cell data were downloaded from Tabula Muris [34] as a Seurat object. Dot plots of Dchs1 gene expression were created for each individual cell type using Seurat’s DotPlot command.

## 3. Results

**Figure 1:**
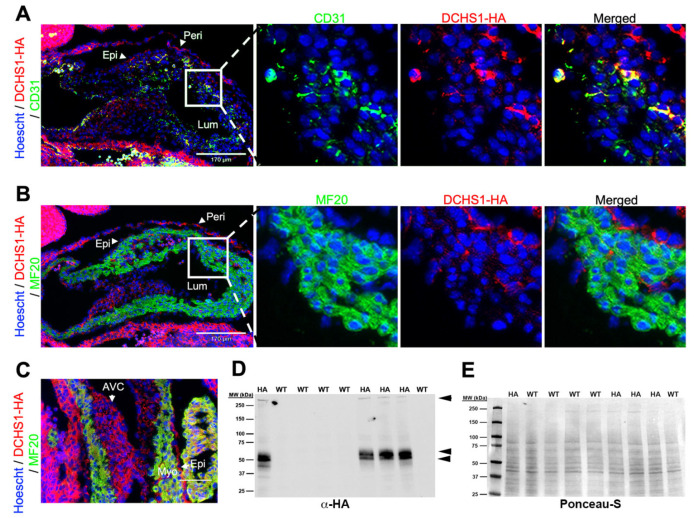
DCHS1-HA expression at embryonic day 11.5. (**A**) IHC of E11.5 embryos for HA (red) and CD31 (green) showing co-localization of Dchs1-HA and a subset of endothelial cells and expression within the pericardium. Scale bar = 170 μm (**B**) IHC of HA (red) and MF20 (green) showing no detectable expression of DCHS1 in cardiomyocytes. Scale bar = 170 μm (**C**) IHC of HA (red) and MF20 (green) in the atrioventricular (AV) cushions showed robust DCHS1 expression as well as expression within the epicardium. Scale bar = 80 μm (**D**) Western analyses of heart lysates at E11.5 probed for HA showing full-length (>250 kDa) and smaller fragments. (**E**) Ponceau-S staining of western blot showing equal loading of proteins. Lumen (Lum), myocardium (Myo), epicardium (Epi), pericardium (Peri), atrioventricular cushions (AVC).

Mice with a targeted insertion of the hemagglutinin (HA) tag in the Dchs1 locus were used to assess endogenous expression throughout embryonic and postnatal life. At embryonic day 11.5 (E11.5), DCHS1-HA expression was observed along the endocardial and epicardial epithelium in the ventricular wall (Figure 1A,B). Prominent expression was also observed throughout the forming valve endocardium and interstitial mesenchyme of the developing atrioventricular (AV) cushions (Figure 1C). Uniform expression throughout the cushion tissue was evident. Confirmation of endothelial expression was observed through co-localization between DCHS1-HA and the pan-endothelial marker (including endocardial cells), CD31, but not detected between DCHS1-HA and the cardiomyocyte marker, MF20 (Figure 1B). By western analyses of whole heart tissue lysate, a high molecular weight band (>250 kDa) was observed, consistent with the predicted size for full-length DCHS1-HA, as well as additional smaller bands around 54 kDa and 50 kDa (Figure 1D).

**Figure 2:**
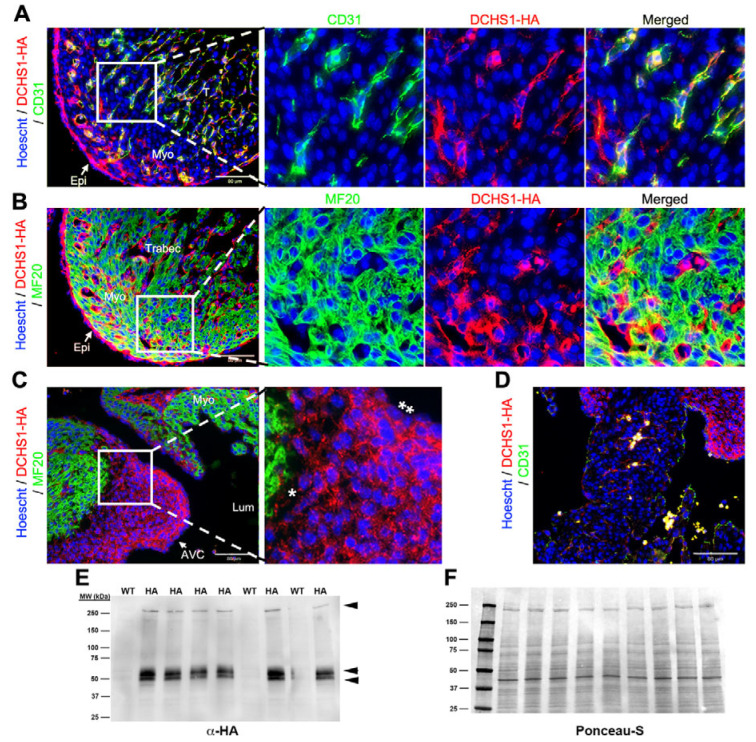
DCHS1-HA expression at embryonic day 13.5. (**A**) Left ventricular free wall cardiac tissue was co-stained with HA (red) and CD31 (green), showing significant overlap (yellow), demonstrating co-expression within endothelial cells. (**B**) No overlap in staining was detected between MF20 (green) and DCHS1 (red). (**C**) Atrioventricular (AV) cushions demonstrated a gradient of DCHS1 expression, with the highest levels within the subendocardium (**) and lower levels closer to the AV myocardium (*). (**D**) Interventricular septum showed relatively low levels of endocardial DCHS1 expression at this time point. (**E**) Western analyses of heart lysates at E13.5 probed for HA showing full-length (>250 kDa) and smaller fragments. (**F**) Ponceau-S staining of western blot showing equal loading of proteins. Lumen (Lum), trabeculated myocardium (T), myocardium (Myo), epicardium (Epi), and atrioventricular cushions (AVC).

By E13.5, DCHS1-HA was more broadly expressed throughout the heart, being present within the endocardium and epicardium (Figure 2A,B) as well as throughout the AV cushions (Figure 2C). Co-localization between DCHS1-HA and CD31 was observed, whereas expression was not detectable in the myocytes (Figure 2A,B). Within the valve endocardium and surface epicardium, polarized expression within these cell types was readily apparent, with expression prominently observed on the basal surface of the cells as well as in junctional interfaces between these cells (Figure 2A–C). At this time point, expression of HA is observed within the AV sulcus, as well as cells that appear to migrate into junctional myocardium from the AV sulcus tissue (Figure 2C). Within the AV cushions, a gradient of expression is observed, with higher intensity of signal in the endocardium and subendocardial mesenchyme, whereas mesenchyme closer to the myocardial wall has much less signal intensity (Figure 2C). Within the myocardial wall, the majority of HA-positive cells co-expressed CD31, indicating that at this developmental stage, DCHS1 protein expression is restricted to the ventricular endothelium (Figure 2A). Notably, staining was most prominent within the ventricular trabeculae (Figure 2A). Limited staining for endocardial expressed DCHS1 was observed in the interventricular septum (Figure 2D). In whole-heart tissue lysate, bands corresponding to full-length DCHS1 and smaller fragments of 50 kDa and 54 kDa are observed, consistent with earlier timepoints (Figure 2E).

**Figure 3:**
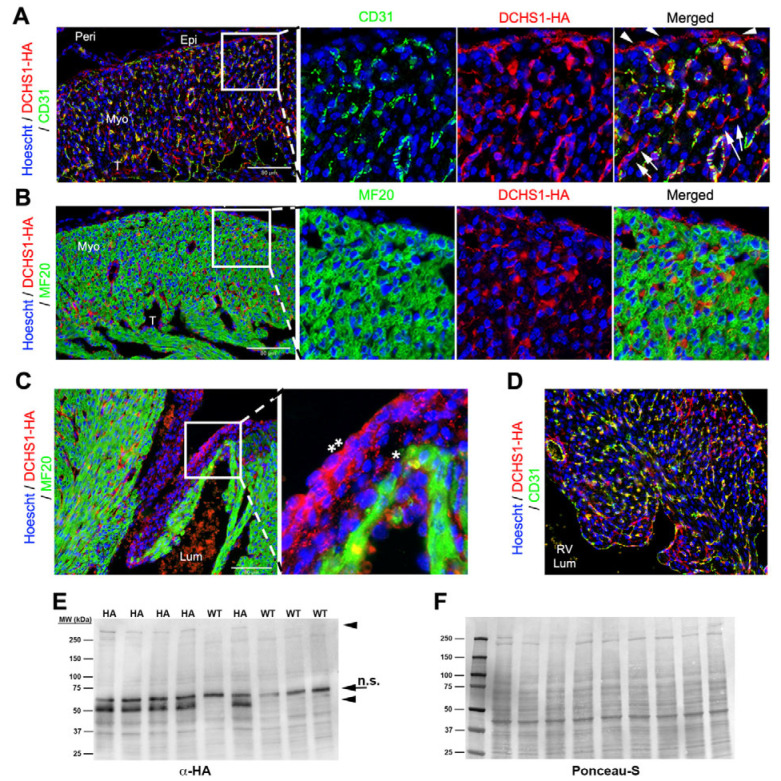
DCHS1-HA expression during fetal gestation. (**A**) Left ventricular free wall cardiac tissue was co-stained with HA (red) and CD31 (green), demonstrating significant overlap. At this timepoint, some non-endocardial, non-MF20 (green) staining is evident (arrows). Epicardial expression of DCHS1-HA is prominent (arrowheads). (**B**) HA (red) and MF20 (green) in the LV free wall showing no detectable overlap in expression. (**C**) IHC for HA (red) and MF20 (green) showing a gradient expression of DCHS1-HA, with the highest levels within the mitral valve subendocardium (**) and lower levels closer to the myocardium (*). (**D**) tissue from the interventricular septum was co-stained with HA (for DCHS1-HA) in red and CD31 (endothelial cell marker) in green. (**E**) Whole-heart tissue lysates of pups from an E18.5 litter were probed blindly for HA (to visualize DCHS1-HA), with observed bands highlighted with arrowheads. Arrow represents non-specific (n.s.) antibody staining on the blot. (**F**) Ponceau-S total protein staining of western blot analyzed in (**E**). Labeled abbreviations: lumen (Lum), right ventricular lumen (RV Lum), trabeculated myocardium (T), myocardium (Myo), epicardium (Epi), and pericardium (Peri).

At E18.5, DCHS1-HA expression was observed throughout the ventricular free wall with dynamic differences based on spatial organization. Although DCHS1 expression is maintained in the epicardium, its intensity appears reduced compared to earlier developmental stages (Figure 3A). Expression becomes more localized to homotypic epicardial junctional interfaces (Figure 3A), in contrast to the broader, basally concentrated pattern observed at earlier timepoints (Figure 1A and Figure 2A). DCHS1 expression is retained in CD31-positive endothelial cells throughout the heart, including within the interventricular septum (Figure 3D). However, at this timepoint, DCHS1 is also detected in a distinct population of cells that lack both CD31 and MF20 staining, suggesting a likely epicardial origin (Figure 3A). These cells are restricted to the compact myocardium and are not observed at this timepoint within the trabeculae layer (Figure 3A). Within this cell type, polarized localization of the DCHS1 is evident, with the highest staining intensity evident in cell bodies protruding towards the ventricular lumen (Figure 3A). Consistent with earlier timepoints, a gradient expression of DCHS1 is observed within the mitral valves (Figure 3C). In whole-heart tissue lysate, bands consistent with full-length DCHS1-HA were observed via western analysis (Figure 3E). At this timepoint, only one smaller band was observed, in contrast to the earlier timepoints, which had identified two bands (Figure 3E).

**Figure 4:**
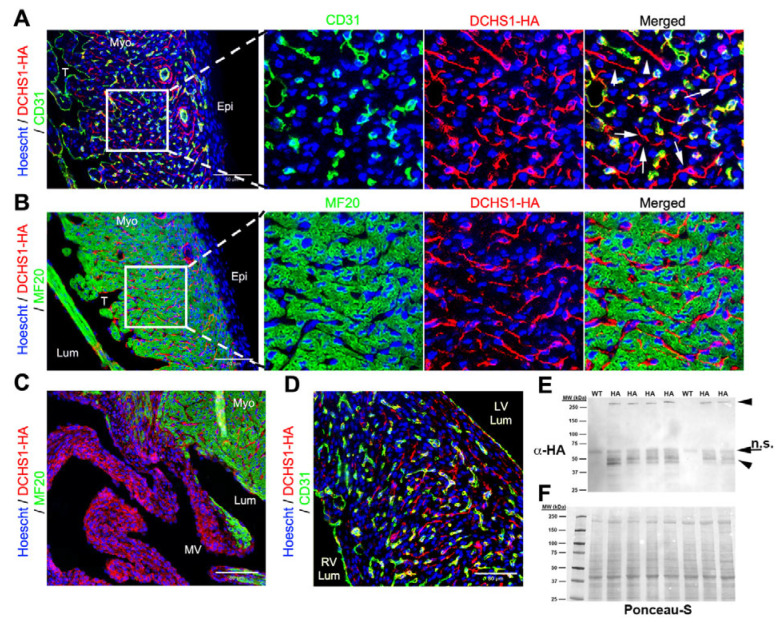
Dchs1 Expression at post-natal day 0 (P0). (**A**) IHC of left ventricular free wall was performed for HA (red) and CD31 (green) or (**B**) MF20 (green). Consistent with earlier timepoints, DCHS1 was not observed on cardiomyocytes but was detected on endothelial cells. Robust and extensive staining was also observed throughout the myocardium in cells that are negative for CD31 and MF20 (indicated by arrows in 1A), likely representing a fibroblast population. (**C**) IHC in mitral valve displayed extensive staining throughout the leaflet, with the most abundant expression within the subendocardial mesenchyme. (**D**) IHC in the interventricular septum was similar with the LV free wall in that DCHS1 is co-expressed with CD31 as well as prevalent in a non-myocyte, non-endothelial cell population. (**E**) Western analyses of whole-heart lysates revealed maintenance of the full-length protein and presence of a small-molecular-weight fragment (arrowheads). Arrow represents non-specific (n.s.) antibody staining on the blot. (**F**) Ponceau-S total protein staining demonstrates equal loading between samples. Lumen (Lum), right/left ventricular lumen (RV/LV, respectively, lumen), trabeculated myocardium (T), myocardium (Myo), epicardium (Epi), mitral valve (MV).

At postnatal day 0 (P0), DCHS1-HA staining was prominent throughout the compact layer of the ventricular free wall but was no longer detectable along the epicardium (Figure 4A). The overall expression pattern resembled that observed at E18.5, with several notable differences. DCHS1-HA staining persisted in CD31-positive endothelial cells; however, these cells now appeared more rounded in morphology (Figure 4A). In contrast, DCHS1-HA-positive cells lacking CD31 and MF20 expression, presumably fibroblasts, exhibited long, slender extensions (Figure 4A). These tendrils were more pronounced than at earlier timepoints and were frequently observed bridging CD31-positive cells, suggesting direct fibroblast–endothelial interactions (Figure 4A and Figure S2). Fibroblast–fibroblast connections were also apparent, with polarized DCHS1-HA-positive processes extending directionally across the compact myocardium (Figure 4A and Figure S2). With the exception of positive endocardial staining, the distal ventricular trabeculae at this stage were mostly devoid of DCHS1 expression, and cardiomyocytes continued to lack detectable DCHS1-HA staining (Figure 4B), consistent with earlier observations. Within the interventricular septum, DCHS1-HA is expressed in CD31+ cells as well as within CD31−/MF20− cells (Figure 4D). Mitral valve expression was throughout the valve and appeared enhanced from fetal timepoints (Figure 4C and Figure S3). Western blot analysis of whole-heart lysates confirmed the presence of full-length DCHS1-HA and the previously reported ~50 kDa band, along with additional smaller bands appearing at this developmental stage (Figure 4E).

**Figure 5:**
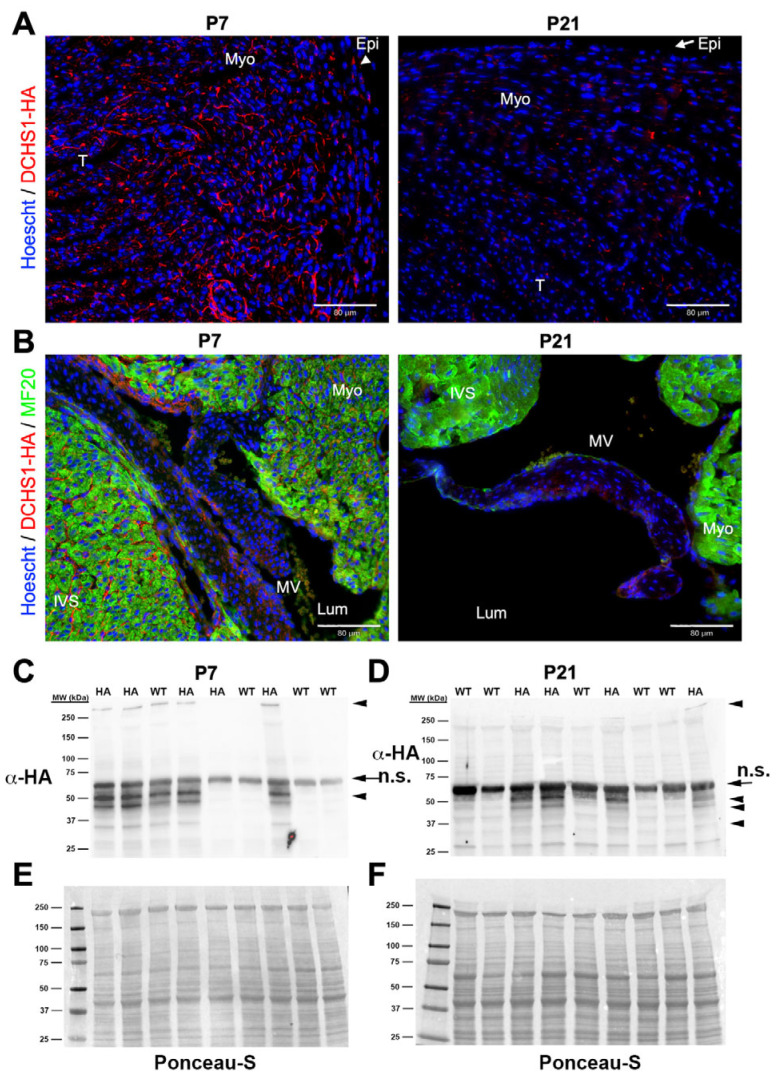
Adolescent and adult Dchs1 expression. (**A**,**B**) Post-natal LV free wall and mitral valves at day 7 (P7) and P21 mice were stained with HA (red) and MF20 (green). (**C**,**D**) Western analyses from whole-heart tissue lysates of pups from P7 and P21 probed for HA. Full length (>250 kDa) was barely detectable at this stage, and reduction in the smaller molecular weight is also apparent at both P7 and P21 (arrowheads). At these timepoints, additional smaller fragments are observed, indicating potential degradation of DCHS1. Arrow represents non-specific (n.s.) immunoreactive bands. (**E**,**F**) Ponceau-S stain showing equivalent protein loading, respectively. Labeled abbreviations: lumen (Lum), trabeculated myocardium (T), myocardium (Myo), epicardium (Epi), mitral valve (MV), and interventricular septum (IVS).

At P7 and P21, DCHS1-HA staining was markedly reduced in left ventricular (Figure 5A) and mitral valve sections (Figure 5B and Figure S3). Western blot analysis of whole-heart lysates revealed bands corresponding to full-length DCHS1-HA at both timepoints, with visibly diminished intensity at P21 (Figure 5C,D). A previously identified unique band near 50 kDa was detected at both P7 and P21. However, lower-molecular-weight bands indicative of DCHS1-HA fragmentation were observed at both P7 and P21, with more pronounced band laddering at P21, suggesting increased proteolytic processing or degradation over time (Figure 5C,D).

**Figure 6:**
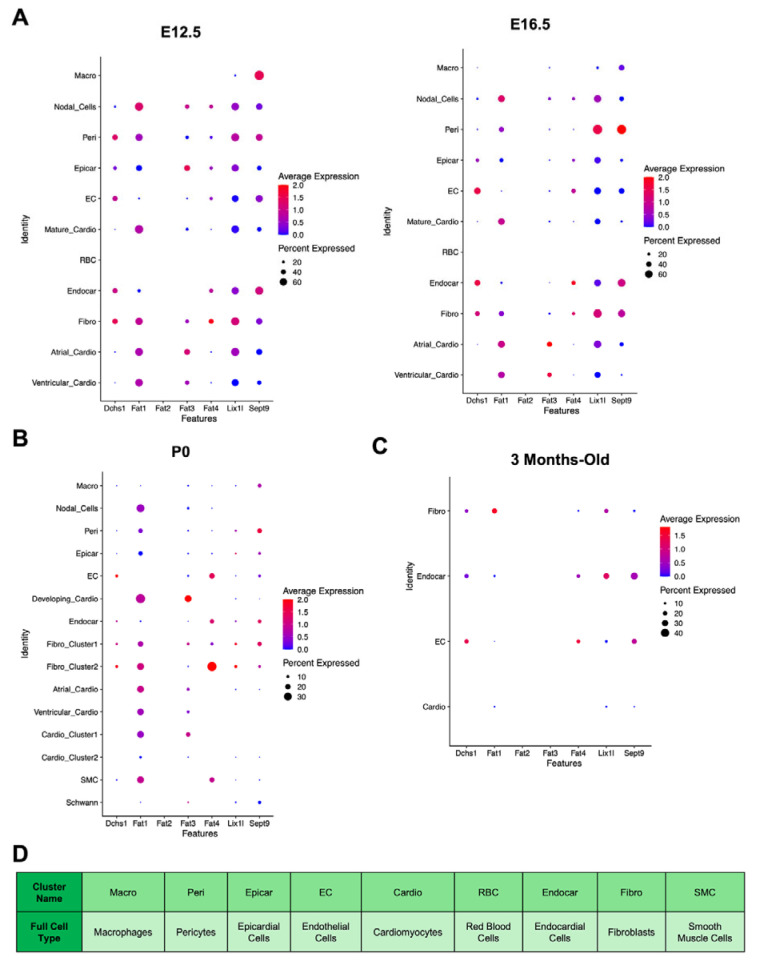
Cell type analyses of mRNAs of Dchs1 and interacting partners throughout developmental and postnatal timepoints. (**A**) Single-cell RNA sequencing data showing mRNA expression distribution of Dchs1, Fat1, Fat2, Fat3, Fat4, Lix1L, and Septin9. (**B**,**C**) Single-nuclei RNA sequencing was performed on post-natal day 0 (P0) and 3-month-old hearts. Fat1 and Fat4 showed significant cellular overlap with Dchs1 profiles, whereas Lix1L and Septin9 showed broader expression in most cell types. (**D**) Legend for panels A–C detailing the full cell type name for any cell cluster labeled with a shorthand name.

Embryonic [32] and adult [34] scRNAseq lookup analysis and snRNA sequencing analysis was used as a second layer of confirmation of Dchs1-cell-type expression throughout cardiac development (Figure 6). Consistent with our immunostaining analyses, Dchs1 mRNA is expressed in non-cardiomyocyte lineages during cardiac development and maturation. At embryonic stages (E12.5–E16.5), Dchs1 mRNA was observed in epicardial, endothelial, endocardial, and fibroblast populations, with transient expression also noted in pericytes at E12.5 that diminished by E16.5 (Figure 6A). In contrast, there was no expression in cardiomyocyte clusters (Figure 6A). In postnatal day 0 (P0) hearts, Dchs1 mRNA remained confined to fibroblasts, endocardial cells, and endothelial cells (Figure 6B). Similarly, in 3-month-old adult hearts, expression, albeit barely detectable by scRNAseq, was restricted to the same cell types, with no detectable expression in cardiomyocytes (Figure 6C).

The expression of Dchs1 binding partners was also examined (Figure 6). Fat4, a known DCHS1 interactor, showed an overlapping expression pattern with Dchs1, being detected in endothelial, endocardial, and fibroblast populations across all stages. Fat1 was broadly expressed in most cell types across development, excluding endothelial cells at P0 (Figure 6B) and macrophages during embryogenesis (Figure 6A). Fat2 mRNA was absent at all stages. Fat3 was specifically expressed in cardiomyocyte clusters and epicardial cells at E12.5, but not in Dchs1-expressing populations (Figure 6A). Lix1l and Sept9, genes implicated in PCP-related signaling and previously shown to interact with the cytoplasmic tail of Dchs1, were broadly expressed in most clusters at all stages, except for red blood cells during embryogenesis and mature cardiomyocytes/nodal cells at P0 (Figure 6B).

## 4. Discussion

This study provides a spatiotemporal analysis of Dchs1 expression during murine cardiac development and maturation, offering new insights into its cellular specificity, developmental regulation, and potential signaling mechanisms. Using an HA-tagged Dchs1 knock-in mouse model, we demonstrate that DCHS1 is robustly expressed during embryogenesis in non-myocyte lineages and is progressively downregulated and likely proteolytically processed during postnatal development.

DCHS1-HA was prominently expressed in the developing atrioventricular (AV) cushions and mitral valve tissue at all developmental stages examined, consistent with previous studies linking Dchs1 mutations to congenital mitral valve disease, including mitral valve prolapse and annular disjunction [17,18,19]. Notably, within the left ventricular free wall, DCHS1-HA consistently co-localized with the endothelial marker CD31 at all developmental stages, confirming expression in endothelial cells. No co-localization was ever observed with MF20, indicating that DCHS1 is not expressed by cardiomyocytes. This non-myocyte-specific expression pattern was corroborated by single-cell and single-nuclear RNA sequencing datasets across embryonic, neonatal, and adult hearts, where Dchs1 transcripts were restricted to endothelial cells, endocardial cells, fibroblasts, and epicardial-derived cells. This is particularly relevant given the array of cardiac defects caused by abnormal Dchs1 expression affecting septation [35], valvulogenesis [17], and mitral annular disjunction [19].

At each developmental timepoint, a band consistent with the expected molecular weight of full-length DCHS1-HA was observed. Two additional HA-specific bands (~54 and ~50 kDa) were consistently observed during embryogenesis, with the 50 kDa form persisting into P21. These fragments likely represent proteolytic cleavage products of the DCHS1 intracellular domain, given that the HA tag was inserted at the C-terminus. While cleavage of DCHS1 has not been previously reported, the detection of fragments and their temporal specificity suggest a previously unrecognized, developmentally regulated cleavage-dependent signaling mechanism. Analogous pathways have been described for other cadherins such as FAT4 [36] and regulatory molecules like NOTCH [37,38], both of which require cleavage to mediate nuclear signaling or morphogenetic control. Notably, the intensity of the lower-molecular-weight bands, presumed to be cleavage products, is substantially greater than that of the full-length DCHS1-HA protein, suggesting extensive proteolytic processing. This likely reflects the release of the extracellular domain from the membrane, leaving only intact full length and the C-terminal fragment detectable by the anti-HA antibody. The loss of the extracellular portion may indicate that DCHS1’s role in mediating cell–cell adhesion, either through homotypic or heterotypic interactions, has been fulfilled. Alternatively, the cleavage may mark a functional transition, enabling the intracellular domain to participate in downstream signaling events or closer subcellular interactions critical for morphogenesis.

Spatially, DCHS1-HA expression follows a developmentally regulated migratory pattern. At E11.5, it is restricted to the epicardial and endocardial surfaces, but by E13.5, it is observed infiltrating the ventricular wall, and by E18.5, extends throughout the full myocardial thickness of the compact myocardium, while sparing the distal trabeculated tissue. This spatiotemporal pattern suggests that epicardial-derived fibroblasts expressing DCHS1 progressively migrate into the myocardium, contributing to structural maturation. Between E18.5 and P0, fibroblast-like DCHS1-HA-positive non-myocyte, non-endothelial cells exhibit polarized extensions that bridge neighboring cells, including CD31-positive endothelial cells. Our data provide molecular evidence of heterotypic interactions between non-myocyte, non-endothelial cells and endothelial cells, as well as homotypic interactions among non-myocyte, non-endothelial cells and among endothelial cells (Figure S2). While the functional consequences of these contacts remain to be fully defined, this represents one of the first demonstrations of coordinated non-myocyte cell–cell connectivity during cardiac development. Such interactions are likely essential for proper myocardial growth, cellular patterning, and tissue organization. Consistent with this concept is the downregulation of Dchs1 protein and mRNA by 3 weeks after birth, demonstrating a primary developmental role for Dchs1 in cardiac morphogenesis.

## 5. Conclusions

This study establishes Dchs1 as a dynamically regulated, non-myocyte-expressed cadherin critical for cardiac development [17,18,19,35], particularly in valve formation and myocardial organization. Dchs1 expression follows a precise spatial and temporal trajectory, originating in the epicardium and endocardium and spreading into the ventricular free wall via endothelial and fibroblast lineages. DCHS1-HA expression is enriched during embryogenesis and sharply reduced after birth, suggesting it plays a time-sensitive role in cardiac morphogenesis. Importantly, we identify cell-type-specific expression overlaps between Dchs1 and Fat4, supporting a conserved DCHS1–FAT4 signaling axis that may operate through homotypic or autocrine mechanisms. The discovery of developmentally regulated cleavage products of DCHS1-HA introduces a novel layer of regulation and potential signaling versatility, akin to other proteolytically activated developmental pathways [11,12,37,38,39]. Together, these findings provide a strong foundation for future mechanistic studies aimed at dissecting the DCHS1-FAT4 signaling pathway in the heart. Understanding this signaling axis may reveal new therapeutic targets for congenital and acquired heart diseases, especially those involving valvular and myocardial defects, conditions already associated with DCHS1 dysfunction in humans.

## Data Availability

Data are available after communication with the senior author.

## Supplementary Materials

The following supporting information can be downloaded at: https://www.mdpi.com/article/10.3390/cells14110774/s1, Figure S1: Knock-in targeting strategy; Figure S2: DCHS1 Endothelial to Non-Myocyte Connections; Figure S3: IHC of DCHS1 throughout development.
