# Supplementary figures and images for: Dynamic Expression and Functional Implications of the Cell Polarity Gene, Dchs1, During Cardiac Development
Source: Cells. 2025 May 24;14(11):774. doi: 10.3390/cells14110774 (PMC12153708; doi:10.3390/cells14110774)

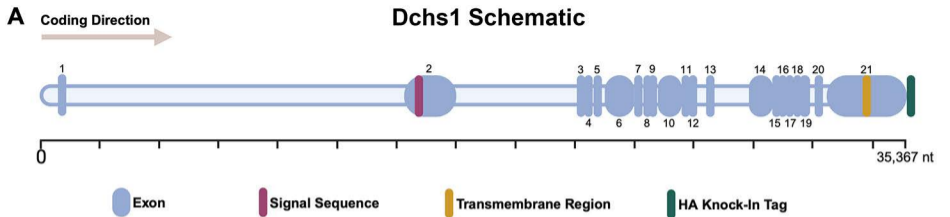

**B**

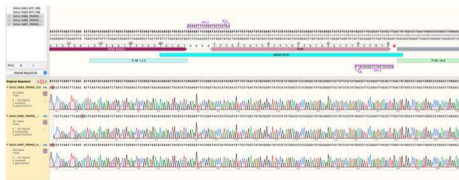

**C**

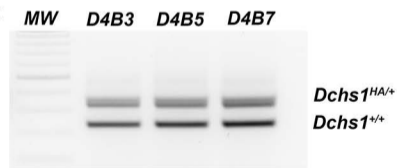

**Figure S1**

Hoescht / DCCHS1-HA  
/ CD31

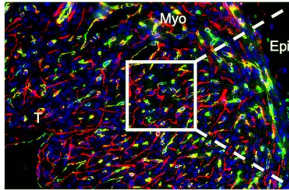

CD31

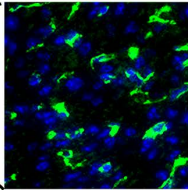

DCCHS1-HA

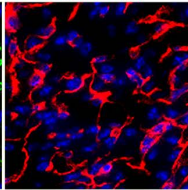

Merged

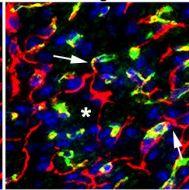

Figure S2

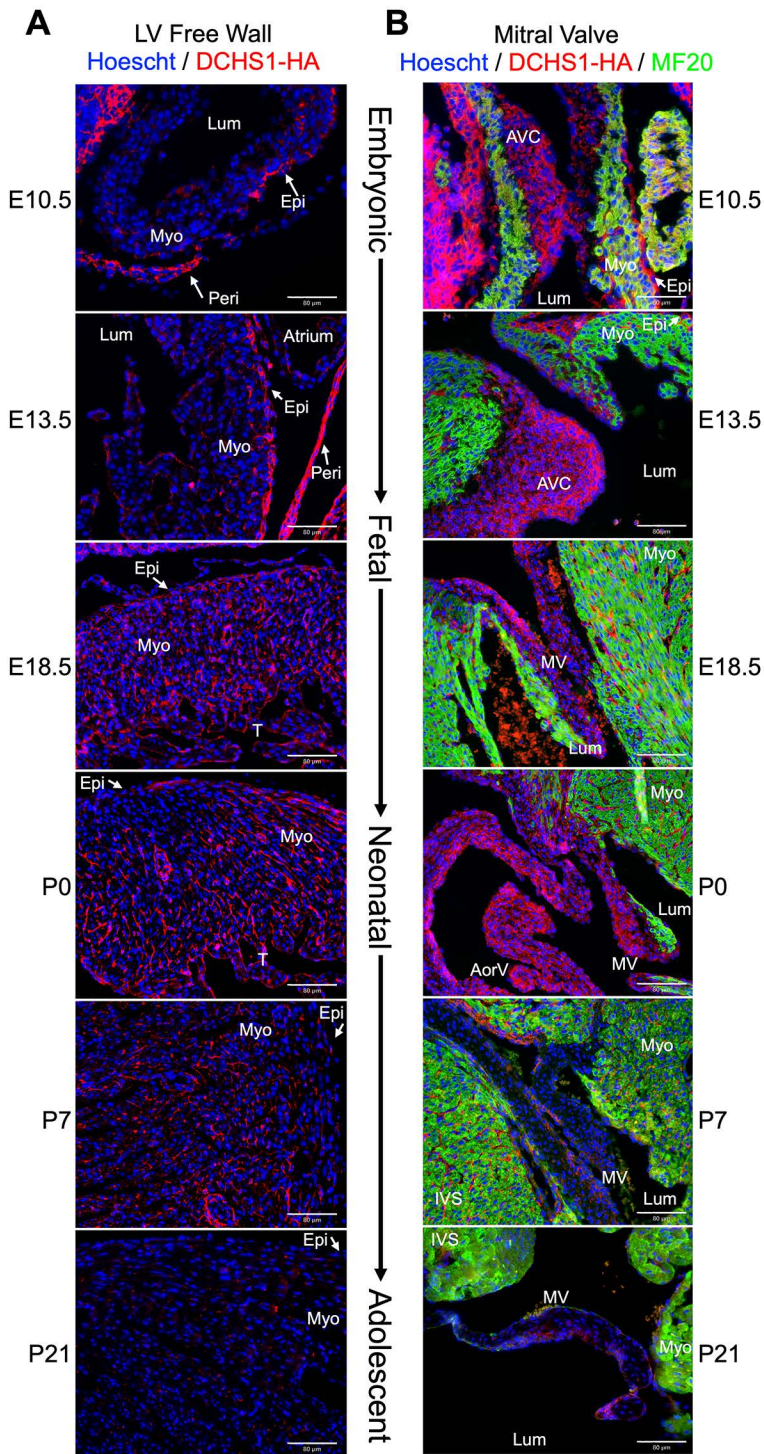

**Figure S3**

Supplement: Supplementary file 1 [file cells-14-00774-s001.zip › cells-3645610-supplementary.pdf]
